# 

**Published:** 1880-02

**Authors:** 


BOOKS AND PAMPHLETS RECEIVED.

**The Physician's Hand-Book for 1880.** By WILLIAM ELMER, M. D., and ALBERT D. ELMER, M. D., New York. W. A. Townsend, Publisher, 1880.

**A Treatise on the Science and Practice of Midwifery.** By W. S. PLAYFAIR, M. D., F. R. C. P. Professor of Obstetric Medicine, in King's College. Third American edition, with notes and additions, by ROBERT P. HARRIS, M. D. Philadelphia: Henry C. Lea. 1880.

